# Brief Report: Above and Beyond Safety: Psychosocial and Biobehavioral Impact of Autism-Assistance Dogs on Autistic Children and their Families

**DOI:** 10.1007/s10803-021-05410-0

**Published:** 2022-01-04

**Authors:** Angela Tseng

**Affiliations:** Department of Psychiatry & Behavioral Sciences, University of Minnesota, 717 Delaware St. SE, Minneapolis, MN 55414 USA

**Keywords:** Autism-assistance dogs, Canine assistance, Service dogs, Psychosocial effects, Chronic cortisol concentration, Parent/child stress

## Abstract

Autism-Assistance Dogs (AADs) are highly-skilled service animals trained primarily to ensure the safety of an autistic child by preventing elopement and mitigating ‘meltdowns’. Although anecdotal accounts and case-studies have indicated that AADs confer benefits above and beyond safety, empirical support anchored in validated clinical, behavioral, and physiological measures is lacking. To address this gap, we studied children and their families before and after receiving a well-trained AAD using a within-subject, repeated-measures design. Notably, this study is the first to assess change in a biomarker for chronic stress in both autistic children and their parents. Final analyses included pre-/post-AAD data from 11 triads (parent/handler-dog-child) demonstrating significantly positive psychosocial and biobehavioral effects of AADs.

## Introduction

Autism spectrum disorder (ASD), a heterogeneous neurodevelopmental disorder (NDD) comprising lifelong challenges in social, communication, and behavioral domains, has reached an unprecedented prevalence estimate of 1-in-54 in the United States (Maenner et al., 2020). Frequently, treatment plans not only need to address core ASD symptoms, but also a variety of co-occurring developmental, psychiatric, neurologic, or medical diagnoses that further impact daily functioning and quality of life (Masi et al., 2017). One approach with the potential to address a number of concerns for autistic individuals and their families is the incorporation of animal-assisted interventions (AAIs)^1^ into home, school, and hospital settings (Dimolareva & Dunn, 2020; Esposito et al., 2011; Johnson et al., 2002); several studies have reported positive effects when human-animal interactions (HAI) have been integrated into ASD therapies (Dimolareva & Dunn, 2020; Droboniku & Mychailyszyn, 2021; Funahashi et al., 2014; OHaire et al., 2013). Anecdotal accounts have also accrued attesting to the benefits of well-trained autism-assistance dogs (AADs) who engage with their human partners on a daily basis. Yet, despite rising interest in the field, the evidence-base for AAIs for ASD remains limited—due, in large part, to considerable variability in research methodologies, implementation, and reporting (Kazdin, 2017; O’Haire, 2013, 2017). Even sparser still are systematic evaluations of whether and how well-trained AADs can impact the lives of autistic children and their families (Butterly et al., 2013).

The primary trained duties of an AAD stemmed from a critical need to prevent child elopement; a foremost concern for many parents of autistic children is that their child may bolt or wander and “expose him or herself to potential danger by leaving a supervised, safe space or the care of a responsible person” (Anderson et al., 2012). One study collected data on missing person cases in the US involving elopement by individuals with ASD across a 5-year period (2011–2016) and reported that, of the 808 cases evaluated, 17% resulted in death, 13% required medical attention, 38% carried a heightened risk of bodily harm (i.e., “close calls), and 1% were still considered missing (McIlwain & Fournier, 2017). Trained AAD teams increase a child’s safety by working as a triad; in public, the child may wear a specially designed belt that connects to the dog’s vest while an adult handler holds the dog’s leash. AADs are taught to resist passively with their body weight if their child attempts to bolt and the tethering system keeps the child with their dog. Caregiver and case study reports have related that AADs can prevent elopement effectively while providing a sense of security for both parents and children (Burgoyne et al., 2014; Burrows et al., 2008). In fact, this trained ability to prevent a child with autism from wandering away confers ‘service animal’ status to AADs, defined by the US Department of Justice as a dog that is individually trained to do work or perform tasks directly related to a person’s disability. Service dogs are permitted to accompany people with disabilities in all areas where members of the public are allowed to go (ADA, 2010).

Another troubling issue affecting families of autistic children is the health and well-being of parents/caregivers who report experiencing higher physiological stress, parenting-related stress, and fatigue than parents of typically-developing (TD) children and children with other NDDs (Baker-Ericzén et al., 2016; Estes et al., 2013; Fecteau et al., 2017; Smith et al., 2009); these experiences may increase parental risk for mental (e.g., anxiety, depression) and physical health (e.g., adrenal, cardiovascular) problems (Foody et al., 2014; Seymour et al., 2012). Myriad factors including child characteristics and behavioral challenges (Olson et al., 2021), as well as sociocultural and economic circumstances (e.g., access to resources, stigma associated with mental health, financial burden of care), can compound to distress parents and affect both child and overall family outcomes by means of transactional pathways (Bonis, 2016; Iadarola et al., 2019; Rodriguez et al., 2019).

Encouragingly, reports of collateral benefits have emerged from families with AADs trained chiefly for safety. One seminal study noted that the contribution of service dogs to family outcomes extended beyond physical welfare to behavioral and psychosocial domains; parents reported that they experienced improved quality of sleep and a greater sense of independence while their children exhibited fewer negative behaviors (e.g., “meltdowns”, “tantrums”, “bolting”) and families overall experienced an increase in social acknowledgement and a decrease in embarrassment or shame in public (Burrows et al., 2008). AADs have also been trained to disrupt potentially harmful repetitive or self-stimulating behaviors as well as provide a modified form of pressure touch therapy practiced by occupational therapists to help autistic individuals reduce levels of arousal and anxiety (Bestbier & Williams, 2017; Grandin, 1992; Krauss, 1987). Further, because simple language is used to work with AADS, children may gain rewarding interactive experiences that then scaffold socialization with other humans (Solomon, 2010). Broadly, these dogs may serve as social catalysts for their human partners by enhancing social interactions, increasing social networks, and reducing instances of social discrimination (Becker et al., 2017; Camp, 2001; Carlisle, 2015; Mader et al., 1989; McNicholas & Collis, 2000).

Yet, while the positive, multidimensional impact of these AADs has been oft reported in anecdotal accounts and case studies, empirical research substantiating these gains is limited. Moreover, documentation is sparse specifying how service dog providers collect outcome data when evaluating the success of their canine placements (Butterly et al., 2013). In order to strengthen the evidence-base for this field, systematic pre-/post-AAD assessments employing validated instruments are warranted. Also, although a handful of studies have been published on the effects of assistance dogs on human psychosocial health and well-being (See Rodriguez et al., 2020 for review), few have focused on dogs trained expressly for ASD. Whereas most adult handler-dog teams (e.g., mobility, seeing, hearing, diabetes) are dyadic, to evaluate the benefits of AADs, we must consider the unique dynamics of the handler-dog-child triad in conjunction with the vast heterogeneity of ASD diagnoses which are often comorbid with other NDDs. Finally, the use of biological measures when possible may provide key objective insights into the long-term effects of having an AAD. To date, however, few studies have included a biomarker measure in their evaluations of AAD success. A review of the extant literature revealed only two such investigations that measured changes in cortisol (salivary), the primary glucocorticoid produced by the activation of the hypothalamic pituitary adrenal (HPA) axis in response to a stressor. Specifically, both studies examined the cortisol awakening response (CAR), a core biomarker of HPA axis regulation related to psychosocial stress and stress-related psychiatric disorders (Fries et al., 2009), and reported decreases of CAR for both parents and children after they receiving trained service dogs (Fecteau et al., 2017; Viau et al., 2010).

The overarching objective of the present study has been to investigate empirically the impact of AADs by collecting psychosocial and biobehavioral data by means of validated instruments designed to better understand the functioning of children and families affected by ASD. In addition to assessment data collected via parent-report (child) and self-report (parent), we included a biological measure of chronic stress in both parents and children to augment our understanding of how AADs may affect physiological health. Chronic cortisol concentrations (CCC) assayed from a single collection of a keratinized matrix (e.g., hair/nails) sample have been shown to represent an accumulation of cortisol secretions over a time frame of months (Meyer & Novak, 2012; Phillips et al., 2021). In contrast, cortisol samples collected from saliva or urine are limited in time (< a few days) and can require repeated measurements across 24-hours over several days to obtain average chronic concentrations (Wosuet al., 2013). Comparative studies examining the correspondence of CCC obtained from scalp-near hair segments to 30-day (3 × daily) average salivary cortisol area-under-the curve levels demonstrated strong associations between CCC and prior 30-day integrated cortisol production measures (Fries et al., 2009; Short et al., 2016). Thus, the use of CCC can also reduce the burden of data collection for participants, particularly vulnerable populations, in addition to providing a gauge of chronic stress retrospectively.

To our knowledge, the present study is among the first to assess CCC in autistic children and the only investigation to examine CCC in both parents and children with ASD. Additionally, no previous reports of the effects of AAD have incorporated CCC measures. Critically, we collected data both before and after participants received their dogs so that we would be able to evaluate outcomes within-subjects. Our study objectives were thus to contribute both quantitative and qualitative data from well-validated instruments to address the question of whether children and their families benefit from these human-canine partnerships across multiple domains.

## Method

All study procedures were approved by the University of Minnesota’s Institutional Review Board and all parents completed informed consent procedures. Participants were informed that their decision to participate would have no bearing on their current or future relationships with the university or the canine training program.

### Participants

Using non-probability, purposive sampling, we recruited families from the top of a regional assistance dog training program’s (Can Do Canines, New Hope, MN, USA) 3–5-year-long waiting list of applications to receive an AAD.

#### Can Do Canines

**Fig. 1:**
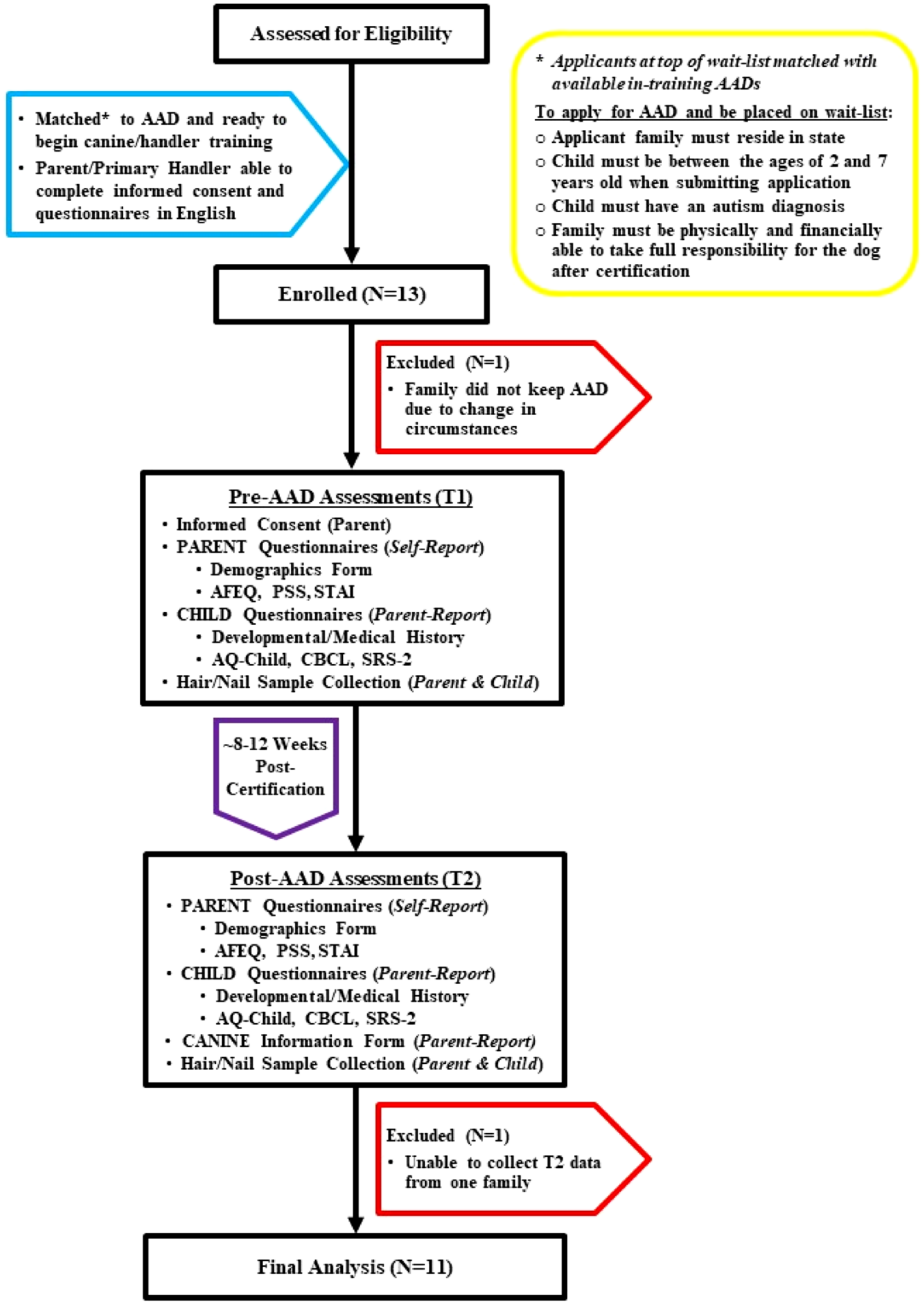
Study-flow diagram

Can Do Canines (https://candocanines.org/) is an internationally recognized, Assistance Dogs International (ADI) accredited, nonprofit organization that trains assistance dogs for hearing loss, mobility challenges, seizure disorders, Type 1 Diabetes, as well as ASD in children. Families are provided with the dogs free-of-charge and the economic burden and time-investment for each certified handler/dog team, combined with the assiduous training and placement standards enforced by the organization, limits severely the number of dogs placed each year. Clients of the assistance dog provider receive AADs whose temperaments/talents were carefully matched to families by highly-experienced trainers. Trainers are able select for certain characteristics (e.g., hypoallergenic breeds) and tailor final training to meet the needs of individual families. To apply for an AAD, children (Ages: 2–7 years when applying) must have a confirmed ASD diagnosis, live within the state, and families must be physically and financially able to take full responsibility for the dog after certification (See Fig. 1 for Study Flow Diagram). An age restriction was established to accommodate the lengthy waitlist and the fact that size must be considered if dogs will be trained to prevent child elopement. By the time they are ready for final training, potential AADs may have already had more than 18 months of socialization, general training, assessments, and intensive training specific to their assistance dog careers. Once the match is made, one caregiver undergoes training to become the primary dog handler and works with trainers and the AAD without their child present. When they are ready to have the dog move into the home, trainers then work with the triad (handler-dog-child) together to build their partnerships and skills in everyday life. These AAD teams require approximately 8–12 weeks to complete team training and certification.

#### Participant Characteristics

Since our potential participant pool was limited to the families who would be receiving an AAD during our period of data collection, our only criteria for inclusion beyond those of the training program were that parents/caregivers be able to provide informed consent and complete questionnaires in English. In total, we enrolled 13 families to participate in the study. Final analyses included data from 11 teams; we were unable to collect post-AAD data from one family and one team experienced a change in family circumstances and had to return their dog. Mean AAD age was 2.9 ± 0.5 years when matched with a family, 45.5% were females, and mean weight was 62.2 ± 7.1 pounds. With the exception of one Standard Poodle, all AADs were Labrador Retrievers, Golden Retrievers, or Labrador/Golden crosses. The designated adult dog handler was the primary parent participant; 100% were mothers, 27% families identified as single-parent households. Secondary parent/caregiver data were collected when possible but were insufficiently powered for further analysis. Formal diagnosis of ASD was confirmed through parent-provided records by the assistance dog organization while additional medical history, including diagnoses of co-occurring neurodevelopmental conditions, was collected via parent-report. All children had a confirmed diagnosis of ASD and 45.5% were non-verbal. Detailed parent and child characteristics are reported in Table 1.

**Table 1:** Participant Characteristics

A. Child		B. Parent (AAD handler)	
Age (years)		Age (years)	
Mean	9.1	Mean	41.3
SD	1.5	SD	4.6
Sex (%)		Sex (%)	
Female	16.7	Female	100.0
Ethnicity (%)		Ethnicity (%)	
Hispanic/Latino	9.1	Hispanic/Latino	9.1
Race (%)		Race (%)	
American Indian/Alaska native	9.1	American Indian/Alaska native	0.0
Asian	9.1	Asian	0.0
Black/African American	9.1	Black/African American	9.1
White/Caucasian	81.8	White/Caucasian	81.8
Other/more than one race	18.2	Other/More than One Race	9.1
Neurodevelopmental disorders (%)		Highest level of education (%)	
Anxiety	90.9	Did not graduate from high school	9.1
Attention-deficit/hyperactivity	36.4	Some college	27.3
Autism spectrum	100.0^a^	College graduate	36.4
Conduct	27.3	Graduate degree(s)	27.3
Global developmental delay	27.3	Annual household income (%)	
Intellectual disability	63.6	$31,000–$40,000	18.2
Motor	18.2	$41,000–$50,000	0.0
Obsessive–compulsive	18.2	$51,000–$60,000	9.1
Seizure	27.3	$61,000–$70,000	18.2
Sleep	72.7	$71,000–$80,000	9.1
Speech and language	45.5	$81,000–$90,000	9.1
		$91,000 +	36.4

^a^Confirmed ASD diagnosis required to apply for AAD

Given that we would not be able to control for heterogeneity in family characteristics and child medical history and treatment, we implemented a repeated measures design that would allow us to examine changes over time within each family. We did, however, ask parents to report ongoing treatments at each assessment and no significant changes in ASD-related treatments between pre-/post-AAD measures were recorded; 36.3% were receiving therapy (e.g., occupational, speech and language, physical, applied behavioral analysis,), 45.5% were receiving therapy and medications, and 18.1% were receiving therapy and “other” treatments (e.g., assistive technology, adaptive sports). We should also note that one common factor amongst the families who chose to remain on the 3–5-year long waitlist for an AAD is a willingness and commitment to bringing an AAD into their lives and the belief that an AAD might be beneficial. Further, families would not likely apply for an assistance dog if their child had known sensory aversions to canines (Grandin et al., 2010) that would preclude meaningful interaction. Applicants were able to make special requests for hypoallergenic breeds but those limitations could lengthen wait-times substantially.

### Study Design

Our assessment battery consisted of parent-report (child) and self-report (parent) questionnaires as well as CCC sample (parent and child) collection. We asked participants to complete pre-AAD (T1) measures after being taken off the waitlist and before receiving their dogs. A follow up assessment (post-AAD; T2) was administered 8–12 weeks after teams were certified. Participants were given the option of completing measures remotely or in-person. Paper questionnaires and consent forms were converted to REDCap (Research Electronic Data Capture), a secure, web-based software platform designed to support data capture for research studies (Harris et al., 2009, 2019). Participants were also given the option to have the researcher collect hair/nail samples in-person or self-collect at home and submit to our laboratory by mail.

Two post-intervention time points were included in the original study design. However, due to institutional research and canine training facility restrictions during the COVID-19 pandemic, we were unable to complete all planned data collection. Moreover, we were concerned that results might be confounded by the considerable stress and changes in routine brought on by the pandemic alongside concurrent civil unrest in our regional community. Consequently, we limited our final data set to teams who completed both of their pre-and post-AAD assessments either before (N = 7) or after Spring 2020 (N = 4). In other words, although we continued to collect follow-up data remotely when possible, we decided to only include data in our final analysis if families completed T2 before March 2020 or if they enrolled after Spring 2020. Ultimately, because the training facility was also required to shut down for a period of time, we did not enroll the next new participant family until October 2020. While participants had been given the option to complete procedures remotely/online before pandemic restrictions were put in place, the latter group of participants were offered the remote/online option only. We report herein on data collected from families before receiving their AAD and 8–12 weeks following team certification.

#### Behavioral/Psychosocial Measures

Behavioral features of children were assessed by having parents complete a pre-/post-AAD battery of questionnaires (see Table 2 for descriptions) including the Social Responsiveness Scale—2nd Edition (SRS-2) (Constantino & Gruber, 2012), the Child Behavior Checklist (CBCL) (Achenbach & Rescorla, 2001), and the Autism Spectrum Quotient—Child (AQ-Child) (Baron-Cohen et al., 2001). In order to gather information about parent/family experiences and concerns, parents also completed the Autism Parenting Stress Index (APSI) (Silva & Schalock, 2012), State-Trait Anxiety Inventory (STAI) (Spielberger, 1989), the Autism Family Experience Questionnaire (AFEQ) (Leadbitter et al., 2018), and the Perceived Stress Scale (PSS) (Cohen et al., 1983). At the second time point, we also asked parents for canine signalment and to respond briefly to some open-ended questions about the AAD’s integration into their household.

**Table 2:** Parent (self-report) and child (parent-report) measures

Name	Description	Retest Reliability	Time (minutes)
*Parent (Self-report)*			
Demographics form	Includes questions about household composition, socio-economic status, family medical history including neurodevelopmental disorders	~ ~	10–15
Autism Family Experience Questionnaire (AFEQ)	48-item questionnaire that assesses family quality of life, includes 4 domains: experience of being a parent; family life; child development and social relationships; child's feelings and behavior	0.83	10
Autism Parenting Stress Index (APSI)	13 items grouped into 3 categories (core social disability, difficult-to-manage behavior, and physical issue) designed to measure aspects specific to families of children with an ASD diagnosis	0.88	5
Perceived Stress Scale (PSS)	10-item instrument that measures degree to which situations in one’s life are appraised as stressful. Items query how unpredictable, uncontrollable, and overloaded respondents find their lives	0.85	5
State-Trait Anxiety Inventory (STAI)	40-item instrument designed to assess levels of state anxiety and trait anxiety; state anxiety defined as a transient momentary emotional status that results from situational stress while trait anxiety represents a predisposition to react with anxiety in stressful situations	0.69–0.89	5
*Child (parent-report)*			
Autism Spectrum Quotient- children’s version (AQ-Child)	50-item parent-report questionnaire designed to measure autism trait severity (4–11 years old)	0.85	10
Child Behavior Checklist/6–18 (CBCL)	113-item questionnaire addressing child’s competencies and problem behaviors, including internalizing and externalizing behaviors	0.80–0.94	15–20
Social Responsiveness Scale, second edition (SRS-2)	65-item rating scale measuring deficits in social behavior associated with ASD, total score reflects social deficit severity with five treatment subscale scores (Social Awareness, Social Cognition, Social Communication, Social Motivation, Restricted Interests & Repetitive Behavior)	0.88–0.95	10–15

AFEQ (Leadbitter et al., 2018); APSI (Silva & Schalock, 2012); PSS (Cohenet al., 1983); STAI (Spielberger, 1989); AQ-Child (Baron-Cohen et al., 2001); CBCL (Achenbach et al., 2001); SRS-2 (Constantino & Gruber, 2012)

#### Biological Measures

To explore AAD impact using a biological measure of chronic stress, we collected samples of scalp hair (posterior vertex) or nail clippings from parents and children for cortisol extraction and analysis by enzyme immunoassay (Cooper et al., 2012; Meyer & Novak, 2012). Although we planned to measure hair cortisol concentration (HCC) only originally, hair collection from some of our initial participants proved to be prohibitively difficult and/or not possible due to lack of scalp hair. Subsequently, participants were also given the option to submit fingernail clippings (Phillips et al., 2021) as an alternative method (Liu & Doan, 2019). Participants provided the same (hair or nail) samples for their pre- and post-AAD measures. Parents were also asked to complete a questionnaire for each hair or nail sample to capture data on hair care and medication use that may affect cortisol assay results (Doan et al., 2018; Hamel et al., 2011). Ultimately, we had to limit our final analysis to the subset of participants from whom we received both pre-/post-AAD samples (Parent, N = 6; Child, N = 5); inclusion/collection of complete datasets was hindered by difficulty with collection, low sample weight, and the presence of steroid medications that may have inflated final concentrations.

### Data Analysis

Using SPSS 25.0 (Statistical Package for Social Sciences, Version 25) we conducted Shapiro–Wilk tests to assess data for normality and Wilcoxon signed-rank tests to assess pre-/post-AAD changes. Both full scale and subscale scores were included when applicable. We used raw scores rather than t-scores for the CBCL and SRS-2 because, at the high end of the distribution, raw scores may be more precise than t-scores (Achenbach & Rescorla, 2001; Constantino & Gruber, 2012). Significance levels were set at alpha = 0.05 (two-tailed). We also examined associations between parent and child data on change in stress and cortisol levels using Pearson correlations.

#### Chronic Cortisol Concentration

We collected 20–50 mg of scalp hair from the posterior vertex region and stored samples at room temperature in dry and dark conditions (Cooper et al., 2012); hair was then wetted with isopropanol, minced into 2 mm pieces, and washed four times with 0.5 mL of isopropanol at room temperature for 30 s to remove external contamination. For fingernail samples, clippings were collected from all ten fingers and then stored and processed using an analogous protocol. Samples were dried under a nitrogen stream and weighed. Cortisol was extracted with 1 mL of methanol overnight at 55 °C, 1 mL acetone for 5 min, and then 1 mL of methanol overnight at 55 °C one more time (Slominski et al., 2015). Pooled solvent fractions were removed under a nitrogen stream. 1 mL of acetone was added and evaporated under a nitrogen stream to chase off the solvents' remnants. Samples were then dissolved in in an assay diluent, randomly distributed on different plates to avoid a batch effect, and analyzed in duplicate using Salimetrics cortisol enzyme-linked immunosorbent assay (ELISA) (Miller et al., 2013). If readings for a sample differed by more than 10% or if readings were too high due to high concentration, the measurements were repeated; also, 5% of samples were randomly reanalyzed to ensure reproducibility.

## Results

**Fig. 2:**
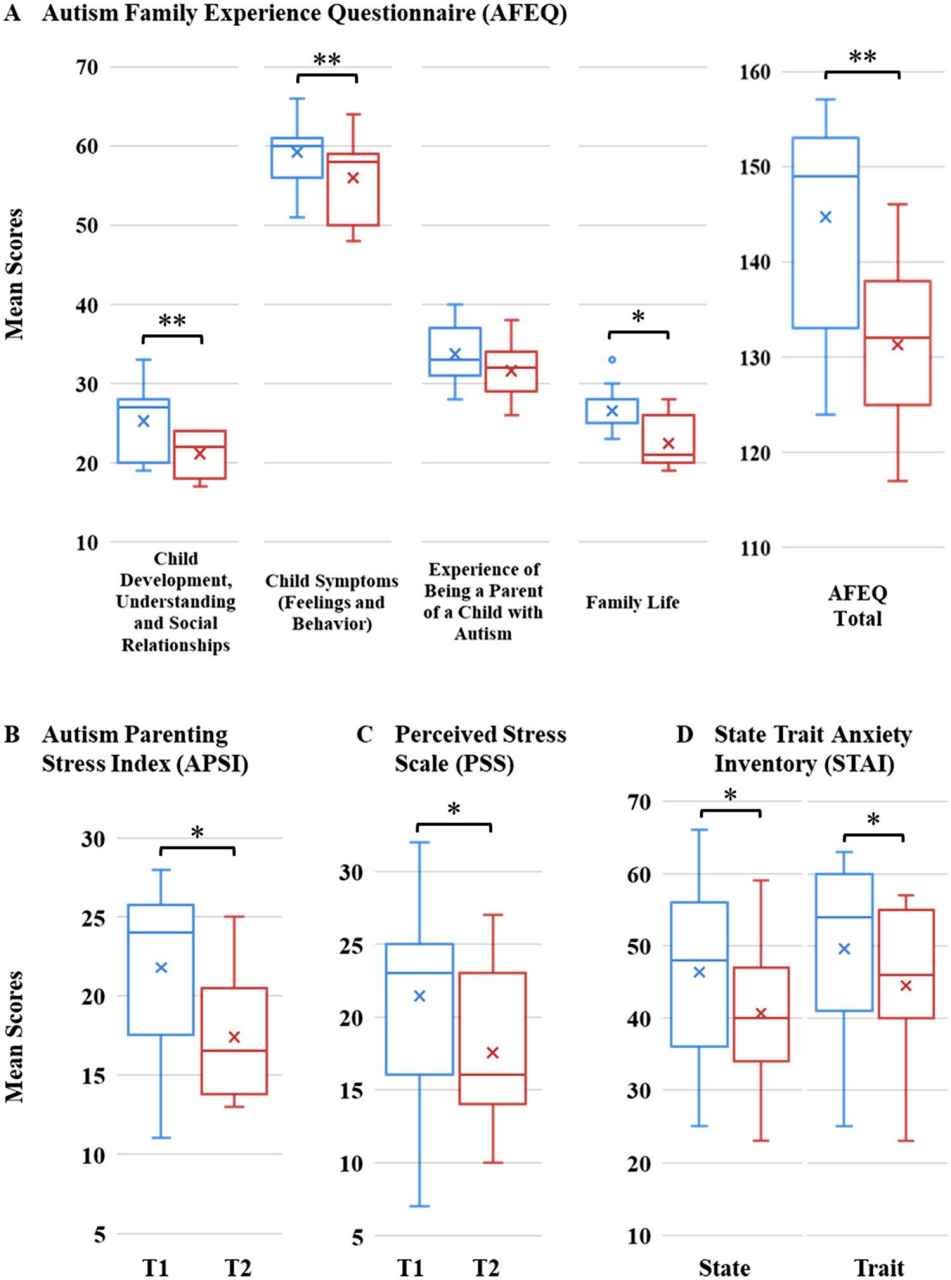
Pre-/Post-AAD mean score differences on parent self-report measures demonstrating: **A** improved family experiences on the AFEQ, **B** reduction of parenting stress on the APSI, **C** reduction of perceived stress on PSS; and **D** reduction of anxiety on the STAI (*p ≤ 0.05; **p ≤ 0.01)

**Fig. 3:**
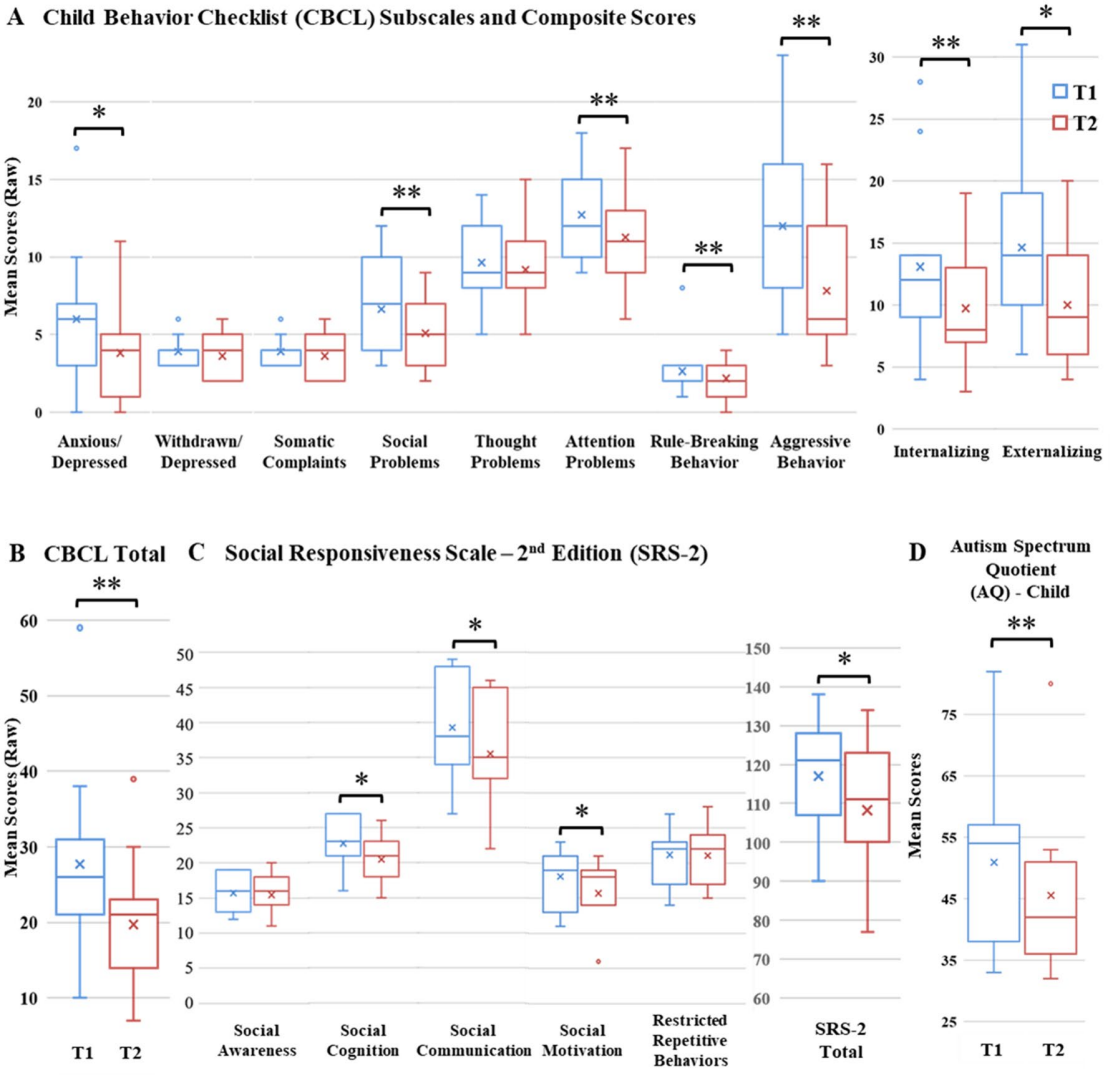
Pre-/Post-AAD mean score differences on parent-report measures demonstrating improvements (decrease in problem scores or reduction in challenges) on the **A, B** CBCL, **C** SRS-2, **D** AQ-Child (*p ≤ 0.05; **p ≤ 0.01)

**Fig. 4:**
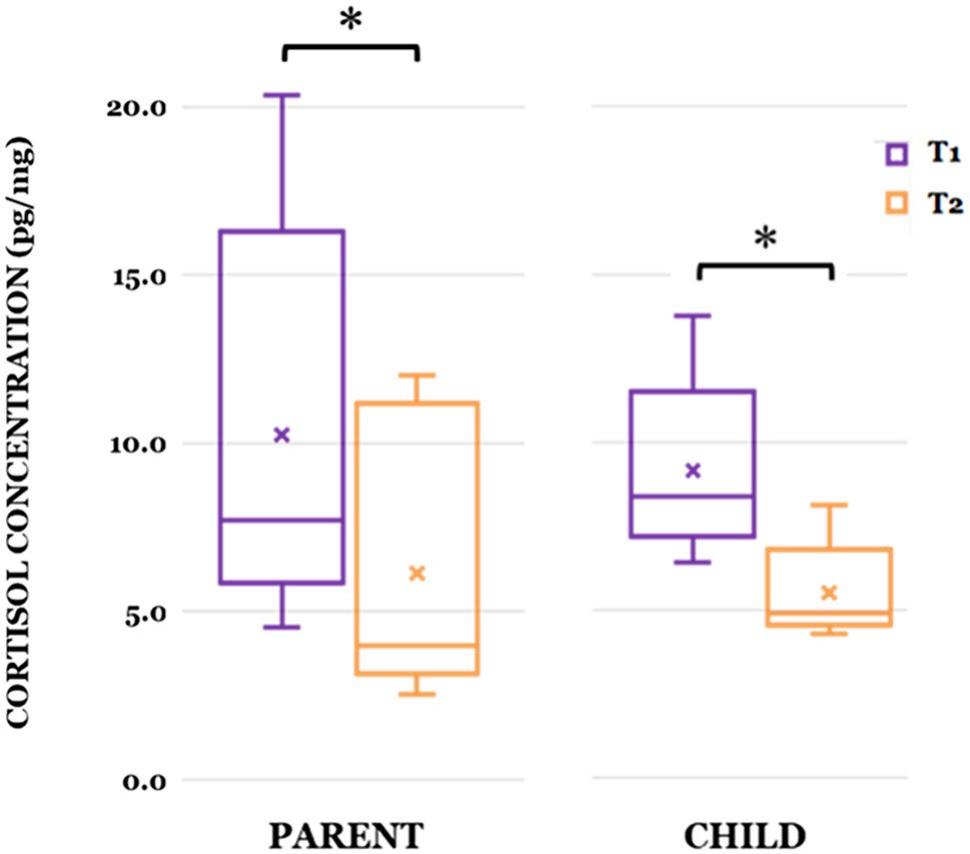
Pre-/Post-AAD differences in chronic cortisol concentration levels for parents and children (*p ≤ 0.05)

Using within-subjects contrasts, we compared measures collected before families received their AAD (T1) and after they had time to complete training and integrate the AAD into their daily lives (T2). Overall, we found significant, positive changes over time for parent, child, and family measures. Complete results are reported in Table 3 and Figs. 2, 3, 4.

**Table 3:** Pre-/Post-AAD results

	Pre-AAD (T1)			Post-AAD (T2)			*Z*^*a*^	*r*	Asymp. Sig. (2-tailed)	Exact Sig. (2-tailed)
	Mean	SD	Median	Mean	SD	Median				
A. Parent (self-report) and child (parent-report) measures										
Parent										
AFEQ (N = 11)										
Child development, understanding, social relationships	50.82	6.76	27.00	44.55	4.72	22.00	− 2.675	0.807	0.007**	0.005**
Child symptoms (feelings and behavior)	35.91	3.05	60.00	34.55	3.27	58.00	− 2.952	0.890	0.003**	0.001**
Experience of being a parent of a child with autism	33.91	3.70	33.00	31.91	3.75	32.00	− 1.636	0.493	0.102	0.109
Family life	27.64	3.32	25.00	24.36	4.20	21.00	− 2.398	0.723	0.016*	0.014**
AFEQ total	148.27	9.55	149.00	135.36	10.11	132.00	− 2.936	0.885	0.003**	0.001**
APSI (N = 10^b^)	21.80	5.81	24.00	17.40	4.33	16.50	− 2.255	0.713	0.024*	0.023*
PSS (N = 11)	21.45	6.96	23.00	17.55	5.43	16.00	− 2.361	0.712	0.018*	0.016*
STAI (N = 11)										
State anxiety	46.36	13.57	48.00	40.64	9.79	40.00	− 2.045	0.617	0.041*	0.043*
Trait anxiety	49.64	12.18	54.00	44.45	10.29	46.00	− 2.398	0.723	0.016*	0.014**
Child										
AQ-child (N = 11)	50.91	13.97	54.00	45.55	13.19	51.00	− 2.503	0.755	0.012**	0.012**
CBCL (N = 11)										
Anxious/Depressed subscale	6.00	4.47	6.00	3.82	3.19	4.00	− 2.273	0.685	0.023*	0.023*
Withdrawn/Depressed subscale	3.91	0.94	4.00	3.64	1.43	4.00	− 0.796	0.240	0.426	0.410
Somatic Complaints subscale	3.18	3.46	1.00	2.27	2.65	1.00	− 1.613	0.486	0.107	0.172
Social problems	6.64	3.01	7.00	5.09	2.39	5.00	− 2.582	0.779	0.010**	0.010**
Thought problems	9.64	2.66	9.00	9.18	2.68	9.00	− 0.621	0.187	0.535	0.580
Attention problems	12.73	3.04	12.00	11.27	3.13	11.00	− 2.676	0.807	0.007**	0.007**
Rule-breaking behavior	2.64	1.91	2.00	2.18	1.40	2.00	− 0.741	0.223	0.458	0.547
Aggressive behavior	12.00	5.31	12.00	7.82	4.00	6.00	− 2.454	0.740	0.014**	0.012**
Internalizing problems	13.09	7.08	12.00	9.73	5.31	8.00	− 2.680	0.808	0.007**	0.004**
Externalizing problems	14.64	6.83	14.00	10.00	4.94	9.00	− 2.315	0.698	0.021*	0.019*
CBCL Total problems	27.73	12.62	26.00	19.73	9.11	21.00	− 2.603	0.785	0.009**	0.006**
SRS-2 (N = 11)										
Social awareness	15.73	2.80	16.00	15.45	2.84	16.00	− 0.051	0.015	0.959	1.000
Social cognition	22.73	3.50	23.00	20.45	3.47	21.00	− 2.149	0.648	0.032*	0.035*
Social communication	39.27	7.67	38.00	35.55	8.38	35.00	− 2.347	0.708	0.019*	0.016*
Social motivation	18.09	4.11	19.00	15.73	5.12	18.00	− 2.363	0.712	0.018*	0.016*
Restricted interests and repetitive behavior	21.18	3.92	22.00	21.09	4.46	22.00	− 0.211	0.064	0.833	0.844
SRS total	117.00	15.92	121.00	108.27	19.20	111.00	− 2.003	0.604	0.045*	0.048*
B. Cortisol concentration measures										
Chronic cortisol concentration (pg/mg)										
Parent (N = 6)	10.255	6.098	7.710	6.127	4.176	3.975	− 2.201	0.898	0.028*	0.031*
Child (N = 5)	9.164	2.774	8.400	5.526	1.518	4.910	− 2.023	0.905	0.043*	0.063†

^a^Wilcoxon signed ranks test: based on positive ranks

^b^Missing data in responses from one Pre-AAD APSI

**p ≤ 0.01

*p ≤ 0.05

†*p* ≤ 0.10

Given the small size of our sample, we employed Shapiro–Wilk tests to assess normality and found that, overall, our data were not normally distributed. Hence, we opted to use non-parametric tests to compare pre- and post-AAD measures. Specifically, Wilcoxon signed-rank tests revealed reductions in levels of experienced and perceived stress on the: PSS, *Z* = − 2.361, *p* = 0.018; APSI, *Z* = -2.255, *p* = 0.024; STAI (State), *Z* = − 2.045, *p* = 0.041; STAI (Trait), *Z* = − 2.398, *p* = 0.016; and the AFEQ (Total Score) *Z* = − 2.936, *p* = 0.003. We also found significant improvements in parent-reports of child behavior and ASD symptomatology: AQ-Child, *Z* = − 2.503, *p* = 0.012; CBCL (Total Problems), *Z* = − 2.603, *p* = 0.009; SRS-2 (Total), *Z* = − 2.003, *p* < 0.045.

We also analyzed CCC levels for both parents and children in the subset of participants who provided both pre- and post-AAD hair/nail samples using Wilcoxon signed-rank tests and found that CCC levels were lower at T2 than at T1 in Parents, *F*(1,5) = 20.852, *p* = 0.006 and Children, *F*(1,4) = 30.600, *p* = 0.005. Inter-plate variability was 2.2% and high, median, low values for final cortisol concentration (%RSD) were 20.35 pg/mg (5.87), 6.85 pg/mg (1.77), and 2.54 pg/mg (0.265), respectively.

For the parent and child dyads with complete cortisol data, we detected a correlation in concentration change (T1-T2) significant at the 0.05 level (1-tailed), *r*(0.822), *p* = 0.044 indicating a reduction in cortisol levels for both parents and children. We also found a significant correlation (1-tailed) for T1-T2 parental cortisol levels and parental PSS scores, *r* (0.814), *p* = 0.047, indicating that reductions in chronic cortisol levels corresponded with reductions in parent perceived stress levels. Also, T1-T2 child cortisol levels were even slightly more correlated (1-tailed) with T1-T2 parental PSS scores, *r* (0.852), *p* = 0.034.

Finally, we asked parents to describe briefly their child’s relationship with their AAD. While we did not collect enough text to conduct thematic analysis, comments were notably positive and highlighted individual differences in each team. Some examples of parental observations are included below.

**Table:** 

Parent 1: *“Child1 takes AAD1 to school every day. The tether system has stopped Child1's elopement. AAD1 can help Child1 calm down when upset and ease his anxiety. Child1's peers like AAD1 so they interact more with Child1 than they did in the past. Child1 feeds AAD1 and will throw a tennis ball for him. Child1 gives AAD1 some instructions but does not initiate a lot of play or petting. Child1 will pet or hug AAD1 when prompted. They sleep in the same room but not the same bed because Child1 does not appreciate AAD1's kisses (licking his face).”*
Parent 2: *“AAD2 and Child2 are best buddies. AAD2 helps Child2 manage his anxiety, stay safe in public places, and allows our family to access our community in a way that we've never been able to do. Awesome!”*
Parent 3: *“You wouldn't necessarily know by interaction how important AAD3 is to [non-verbal] Child3 but Child3 loves AAD3 and he is very important to him. AAD3 sleeps with Child3 and assists when we are out in the community.”*

## Discussion

Our primary study objective was to assess the multidimensional impact of well-trained AADs on autistic children and their families across key domains of function. By recruiting from the top of a wait-list for AADs, we were able to enroll participants shortly before they received their dog, thus allowing us to collect pre-/post-AAD data using a battery of psychosocial and biobehavioral assessments. Our findings provide substantive support for the positive effects of AADs above and beyond their duties as a child’s “sentinel of safety” (Burrows et al., 2008).

The observed benefits of AADs may not be surprising since young children, as early as 9-months of age, have demonstrated an attraction to animals, often preferring them to inanimate objects (DeLoache et al., 2011; Kahn, 1997; Lobue et al., 2013; Ricard & Allard, 1993). Positive interspecies interactions have also been associated with increased concentrations of oxytocin and decreased cortisol levels in both humans and canines (Handlin et al., 2015; Nagasawa et al., 2015; Odendaal & Meintjes, 2003). During medical procedures, the presence of a companion animal has been shown to reduce a child’s physiological arousal and behavioral distress (Nagengast et al., 1997; Vagnoli et al., 2015). Correspondingly, during a laboratory-based stressor, rise in perceived stress for TD children (7–12 years) was buffered significantly by the presence of the family pet dog, relative to children who were alone or with a parent (Kertes et al., 2017). As invaluable sources of socio-emotional support (Melson, 2003), animals may also serve as transitional objects, through which children can transfer their established bonds to humans (Martin & Farnum, 2002). For children especially, dogs provide multisensory experiences and direct feedback in the context of nonverbal actions that may be more easily deciphered at early developmental stages (Prothmann et al., 2009; Redefer & Goodman, 1989).

Prior research has suggested that dogs are particularly adroit at eliciting prosocial behavior, acting as social catalysts with humans, as well as reducing physiological arousal and stress in children and adults (Fecteau et al., 2017; McNicholas & Collis, 2000; Viau et al., 2010). Consistent with these findings, our data show significant pre-/post-AAD improvements for children on the AQ-Child, the CBCL (CBCL Total Problems; Anxious/Depressed, Social Problem, and Attention Problem Subscales; Internalizing and Externalizing Problem Composites), and the SRS-2 (SRS Total; Social Cognition, Social Communication, and Social Motivation Subscales). Parents self-reported significantly reduced stress and anxiety on the APSI, PSS, and STAI (State and Trait) and significantly improved family experiences overall on the AFEQ (AFEQ Total; Child Development, Understanding, & Social Relationships; Child Symptoms—Feelings & Behavior; Family Life Subscales). Both parents and children with pre-/post-AAD CCC data showed a reduction on our objective physiological measure of chronic stress. However, while the majority of outcome measures indicated significant pre-/post-AAD improvements, it is worthwhile to consider those areas that yielded trend improvements on the AFEQ (Experience of Being a Parent of a Child with Autism Subscale, *p* = 0.102) and the CBCL (Somatic Complaints Subscale, *p* = 0.107) and those measures that returned non-significant results on CBCL Subscales (Withdrawn/Depressed, Rule-Breaking Behavior, Thought Problems) and SRS-2 Subscales (Social Awareness, RRBs). By differentiating between domains that are more or less susceptible to the presence of an AAD, we may be afforded insight into the potential mechanisms of actions subserving the dynamic, ongoing relationships within parent/handler-dog-child triads.

In evaluating how the integration of a well-trained AAD can result in long-term changes in the lives of autistic children and their families, adopting a dynamic biopsychosocial perspective may be useful to contextualize the role of AADs (Gee et al., 2021; Lehman et al., 2017). Within this framework, the AAD’s role in preventing a child’s elopement may be construed as a continuous interplay between biological, psychological, and social factors within a non-static environment. For example, by consistently and effectively preventing a child from eloping, the AAD helps alleviate some of the acute safety concerns reported by parents/caregivers of autistic children (Bonis, 2016; Burrows et al., 2008; Rodriguez et al., 2019). Over time the increased sense of security and social acknowledgment afforded by the AAD may reduce chronic physiological and psychological stress in parents, improving overall quality of life for the family (Eddy et al., 1988; Mader et al., 1989). Further, parents have reported that having the AAD to support their child enables them to go on family outings, feel more independent, and be more connected socially, processes that can also serve to augment mental health and well-being more broadly (Burgoyne et al., 2014; Smyth & Slevin, 2010).

### Limitations

While findings from this investigation provide significant support for the benefits of AADs, the data are limited in a number of ways.

First, we must note the conclusions drawn from these AAD-teams should be considered in view of their highly-specialized training and stringent certification criteria and may not be generalized across animals described as emotional support, therapy, comfort, or companion animals who have not received comparable levels of training.

Second, we did not include a wait-list control group (families who applied for an AAD but did not receive a dog during the same period of time) or a non-wait-list control group (families from the community who had not applied for an AAD). Given the highly multifactorial nature of each family’s individual characteristics, the unpredictable length of time each family might be on the wait-list, and the limited number of AADs available, we decided to constrain the study to a single group, repeated measures design. The additional variability introduced by families who had not applied for an AAD (non-wait-list controls) would render comparison data even more difficult to interpret. Additionally, including a control group from further down the wait-list would require participant families to remain on the wait-list for the duration of the study collection period and we did not wish to interfere with standard operating procedures of the training program. In particular, we did not want study participation to be a factor if an AAD candidate proved to be a good match for a control-family as collecting an appropriately-timed T2 assessment would delay the process of getting the AAD team started. Moreover, families unlikely to receive a dog during our collection period (i.e., bottom of the 3–5 year wait-list) would include a younger cohort of autistic children who would be poorly matched to the active group if we implemented a cross-sectional design. Further, families on the wait-list could not be restricted from introducing, discontinuing, or modifying therapies/medications during the study period; yet, these alterations would inexorably confound comparisons to the active group. While families who did receive an AAD were also not constrained from altering their treatment plans, the training process of becoming an AAD team is quite involved and we surmised that families would not have the time to modify their existing treatment plans substantially; we did not note any significant alterations in child therapies/medications pre-post-AADs in our sample but we could have factored in those changes to our final analysis as needed.

Third, due to the high demand and low supply of qualified AADs, our sample size was expectedly quite small. In anticipation of this limitation, we chose a within-subject design to examine pre-/post-AAD changes for each family; we were able to demonstrate significant, quantifiable changes from T1 to T2. Also, because we were unable to collect all data from the third time point as originally planned, we were precluded from gauging if improvements were maintained long-term. Additional data points could have provided insight into whether continued interaction with AADs would lead to sustained and/or greater/fewer changes over time. For example, while we posit that some AAD effects follow a more protracted time course through indirect pathways, these may not be evident until more time has passed. One putative mechanism entails the proximal reduction of physiological arousal stress and an increase in feelings of physical safety with the AAD that may impact sleep quality distally in time. Several parent participants reported that their children have difficulty sleeping which, in turn, affected their own sleep quality. Sleep deprivation indubitably plays a role in mental health and well-being, which can then impact multiple levels of family systems and behavior (Mihaila & Hartley, 2018). However, several families noted that with the AAD’s presence, their children began sleeping through the night, perhaps due to an increased sense of security or their canine’s de-arousing capabilities.

Next, we could not control for the myriad variables that may have contributed to changes over the study timeline. For example, we cannot rule out the impact of developmental change over the study months and families maintained their ongoing treatment and medication schedules while participating. Although our T2 data demonstrated significant improvements in participants relative to their T1 data, we cannot be certain that changes were not due to variables such as maturation, concurrent treatments, or unknown environmental factors. Also, most measures were parent-report and parent-self report assessments, which may be subject to response bias. Yet, given that the autistic children in our study were quite young, heterogeneous in their presentation, and all had co-occurring challenges (e.g., non-verbal, co-morbid NDDs), it was not feasible to administer an objective task-based or observational measure that would be developmentally appropriate for all participants. We were, however cautiously selective in the measures chosen for inclusion in our assessment battery; all instruments were validated, reliable (see Table 2), behavioral and psychosocial instruments that have been developed for and used widely to evaluate child functioning and development.

Finally, we experienced difficulty when collecting samples for cortisol assay because several participants had very short or no scalp hair. Similar issues when collecting fingernail samples arose because some individuals bit their fingernails or kept their nails quite short. An alternative option that may offset some of these issues in future studies might be the use of toenail clippings to ascertain cortisol concentration. Overall, considering the heterogeneity of our participant families, we are reasonably confident that receiving an AAD, the one consistent change for all families during the study collection period, was a driving factor in positive outcomes. Nevertheless, findings based on our limited sample size must be interpreted with caution.

## Conclusions

To our knowledge, the present study is the first to examine psychosocial and biobehavioral effects of assistance dogs trained specifically for ASD using validated and standardized measures of family experience, parental stress, autism symptom severity, and child behavior; these data are also the first to evaluate a biological marker for chronic stress in both children and parents/caregivers. Our findings augment significantly our evidence-base for the benefits of AADs on autistic children and their families across multiple domains.

At present, well-trained assistance dogs, particularly those for ASD, remain a highly limited ‘commodity’, requiring considerable, often prohibitively high, investment of resources by families and service dog providers. Average wait times for well-trained AADs can exceed 3 years and the estimated total cost to raise and train just one dog can surpass $55,000 (Cooper, 2021; Ensminger, 2010; Konrad, 2009). Further, after team certification, families must assume all financial responsibilities for canine care. Currently, no health insurance policies cover any of these expenses beyond the possible application of pre-tax healthcare accounts, (Internal Revenue Service, 2020), and most service dog providers require that families contribute at least part of the costs themselves. Additionally, while US federal law mandates access for service animals to all public areas including schools, the Americans with Disabilities Act also requires that the animal be under the handler's control at all times (ADA, 2010). However, because public facilities are not themselves responsible for service animals, schools do not have to provide handlers. AADs are trained to work as part of a triad, and unless an adult dog-handler is available, the child is still prohibited from bringing their AAD to school. Given that these considerable financial and regulatory barriers remain, further work is needed to broaden the scope of our research to more service dog providers and autistic individuals both within the US and internationally. An enhanced understanding of factors contributing to the effectiveness of AADs will serve to refine canine placement procedures and training approaches, with the ultimate goal of increasing availability and accessibility of AADs for families who may benefit substantially from these specialized human-canine partnerships.

## Footnotes

1 For the sake of clarity, the term “animal(s)” will be used to signify “non-human animal(s)” throughout the manuscript.
